# Refractoriness to transarterial chemoembolization in patients with recurrent hepatocellular carcinoma after curative resection

**DOI:** 10.1371/journal.pone.0214613

**Published:** 2019-04-04

**Authors:** Mi Young Jeon, Hye Soo Kim, Tae Seop Lim, Dai Hoon Han, Beom Kyung Kim, Jun Yong Park, Do Young Kim, Sang Hoon Ahn, Gi Hong Choi, Jin Sub Choi, Kwang-Hyub Han, Seung Up Kim

**Affiliations:** 1 Department of Internal Medicine, Institute of Gastroenterology, Yonsei University College of Medicine, Seoul, South Korea; 2 Yonsei Liver Center, Severance Hospital, Seoul, South Korea; 3 Department of Surgery, Yonsei University College of Medicine, Seoul, South Korea; Texas A&M University, UNITED STATES

## Abstract

**Background/Aims:**

It is important to identify patients who are refractory to transarterial chemoembolization (TACE), which is performed for the treatment of hepatocellular carcinoma (HCC). We investigated the predictors of poor treatment outcomes in patients with recurrent HCC treated who were treated with TACE after curative resection.

**Methods:**

428 patients with recurrent HCC after curative resection who were treated with TACE were enrolled.

**Results:**

The median age of the study population was 59.2 years. On multivariate analysis, ≥2 TACE procedures within 6 months (hazard ratio [HR] = 1.898), and the des-gamma carboxyprothrombin level (HR = 1.000) independently predicted the progression to Barcelona Clinic Liver Cancer (BCLC) stage C in patients with BCLC stage 0-B HCC (both *P*<0.05). In addition, ≥2 and ≥3 TACE procedures within 6 months independently predicted mortality in the entire study population (HR = 1.863 and 1.620, respectively). The probability of progression to BCLC stage C in patients with BCLC stage 0-B HCC and the mortality rate in the entire study population were significantly higher in patients treated with ≥2 TACE within 6 months than in those who underwent fewer procedures (*P* = 0.002 and *P*<0.001, respectively).

**Conclusions:**

More than 2 TACE procedures within 6 months might be associated with the refractoriness to TACE in patients with recurrent HCC after curative resection.

## Introduction

Hepatocellular carcinoma (HCC) is ranked as the 5th most common cancer worldwide, and the 3rd leading cause of death [1, 2]. Although ultrasound-based surveillance for HCC is applicable in clinical practice, HCC is still diagnosed in the late stage in a significant proportion of patients, for which only palliative treatments such as transarterial chemoembolization (TACE), targeted systemic or hepatic arterial infusion chemotherapies are available. However, if HCC is detected in the early stage, several treatment options such as liver transplantation, surgical resection, or ablative therapy can be attempted with a curative intents [3].

Hepatic resection has been considered the mainstay of curative treatment for HCC, and offers a significantly better survival [4–6]. However, due to the high risk of recurrence despite curative resection, the long-term outcomes of patients with HCC who underwent resection remain unsatisfactory [7]. In contrast to the ultrasound-based surveillance of patients without HCC, the intensive follow-up strategy with dynamic imaging modalities, such as computed tomography (CT) or magnetic resonance imaging (MRI) after HCC resection has increased the possibility of detecting HCC recurrence at the early stage. However, re-resection is not always feasible because of technical difficulties such as postoperative adhesion, insufficient remnant liver volume, or patient refusal [8]. Thus, TACE has been frequently performed to control recurrent HCC after resection, if there is no evidence of extra-hepatic metastasis or vessel invasion [9–11].

On the basis of the reported survival benefit of TACE, TACE has been widely used for patients with Barcelona Clinic Liver Cancer (BCLC) intermediate stage HCC [10, 12, 13]. However, recent studies have shown that repeated TACE sessions within a short period due to incomplete response or appearance of new lesions can cause stage progression or the deterioration of liver function even for intermediate-stage HCC [14].

When only targeted systemic therapy for patients with stage-migrated HCC or conservative managements for those with deteriorated liver function are available, poor survival is expected [3, 15]. However, molecular target or immunotherapeutic agents can be considered, when the poor outcomes are expected after repeated TACEs in patients with multiple tumors, major vessel invasion, hypovascularity, or borderline liver function. Thus, it might be important to identify patients at high-risk for TACE refractoriness and set up the optimal timing to switch treatment modality, because early detection of TACE refractoriness would enable early amendments in the treatment strategy and better survival outcomes [14]. Indeed, several studies have shown that the early switch from TACE to sorafenib significantly prolonged overall survival when compared with adherence to repeated TACEs among patients with TACE refractoriness [15].

Although TACE has been frequently performed in patients with recurrent HCC after curative resection and the concept of TACE refractoriness has been previously demonstrated, it is not known whether the term “TACE refractoriness” is applicable in patients with recurrent HCC after curative resection. Thus, we aimed to identify the predictors of stage progression and mortality during repeated TACE sessions, and to define TACE refractoriness in patients with recurrent HCC after curative resection.

## Materials and methods

### Patients

**Fig 1:**
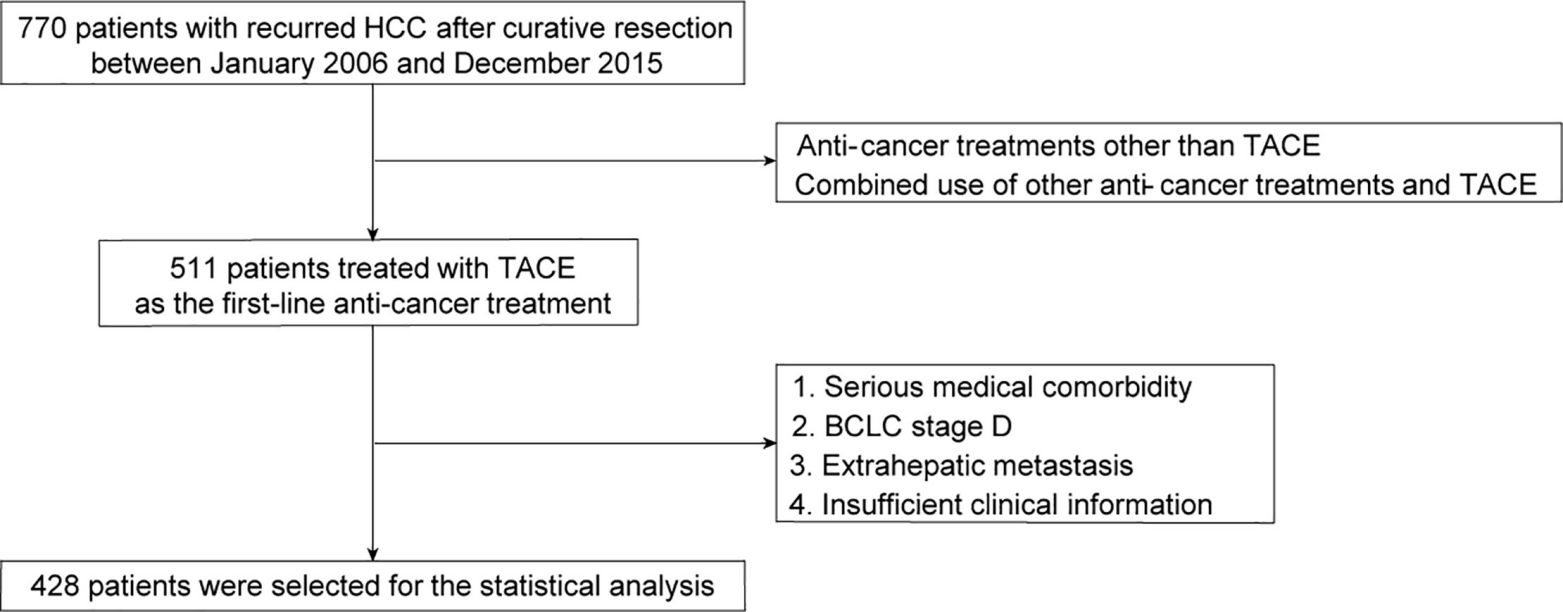
Flowchart of the study population. A total of 770 patients with hepatocellular carcinoma (HCC) underwent curative resection between 2006 and 2015 in our training cohort. After the application of exclusion criteria, the training cohort consisted of 428 patients who underwent transarterial chemoembolization because of recurrent HCC after curative resection.

Through a retrospective review of the prospectively registered data bank of Yonsei Liver Center, Severance Hospital, Yonsei University College of Medicine, Seoul, Korea, eligible patients with recurrent HCC after curative resection between 2006 and 2015 were considered for inclusion in this study. The exclusion criteria were as follows: 1) anti-cancer treatments other than TACE; 2) combined use of other anti-cancer treatments and TACE; 3) serious medical comorbidity; 4) BCLC stage D; 5) extrahepatic metastasis; and 6) insufficient clinical information (**Fig 1**). All relevant data are available with Supporting Information files (**S1 Dataset**).

The study protocol was in accordance with the ethics guidelines of the 1975 Declaration of Helsinki and the study procedure was approved by the institutional review board of Severance Hospital. The need for written informed consent was waived because of the retrospective nature of the study.

### Diagnosis and staging of HCC

According to the guidelines proposed by the Korea Liver Cancer Study group [16], HCC is diagnosed if patient has 1 or more risk factors (chronic viral hepatitis or cirrhosis) and any 1 of the following: 1) serum alpha-fetoprotein (AFP) level >400 ng/mL and at least 1 positive finding for typical HCC on dynamic CT, MRI, or hepatic angiography; 2) serum AFP <400 ng/mL and at least 2 positive findings for typical HCC on those imaging studies. A positive finding for typical HCC on dynamic CT or MRI was defined as increased arterial enhancement followed by decreased enhancement compared with the liver (washout) in the portal or equilibrium phase [16–18].

### TACE procedure

TACE was performed through selective infusion of a mixture of iodized oil contrast medium (Lipiodol; Guerbet, Aulnay-sous-Bois, France) and doxorubicin (Adriamycin; Ildong Pharmaceutical, Seoul, Korea) followed by embolization of feeding arteries using gelatin sponge particles (Cutanplast; Mascia Brunelli S.p.A., Milan, Italy). Embolization was performed until stasis was achieved in the second- or third- order branches of the right or left hepatic arteries [19]. TACE was repeated on an “on-demand” basis at 6- to 8-week intervals when a residual viable tumor or new intrahepatic lesions was detected in the liver on a follow-up assessment, in the absence of extrahepatic metastases, major portal vein invasion, or deterioration in clinical status or laboratory values [20].

### Assessment of treatment responses

HCC staging and response evaluation at the time of recurrence or after TACE was done by radiologists who were aware of treatment history of TACEs. Treatment responses were assessed 4 weeks after the initial TACE by using a modified Response Evaluation Criteria in Solid Tumors (RECIST) guideline [3, 19, 21]. The modified RECIST guideline defines viable tumors according to uptake of contrast material in the arterial phase of dynamic CT or MRI; tumors retaining iodized oil and necrotic lesions without intratumoral arterial enhancement were considered necrotized tumor foci.

### Statistical analysis

The baseline characteristics of the patients are expressed as median (interquartile range, or n (%), as appropriate. Independent t-test (or Mann-Whitney test) and chi-square test (or Fisher’s exact test) were used to compare the characteristics between the subgroups with early (≤ 2 years) and late (> 2 years) recurrence of HCC after curative resection [22]. Univariate and multivariate Cox regression hazard models were used to produce crude and adjusted hazard ratios (HRs) and their 95% confidence intervals (95% CIs) for potential risk factors. Variables with a *P* value of <0.05 in univariate analyses were included in the multivariate models. A *P* value of <0.05 was considered statistically significant. The cumulative stage-progression free-survival and overall survival rates were estimated using the Kaplan-Meier method, and median survival times and their 95% CIs are reported. The log-rank test was used to assess and compare the survival differences between the groups. All statistical analyses were performed using SPSS 23.0 for Windows (IBM Corp., Armonk, NY, USA).

## Results

### Patient characteristics

A total of 770 patients with recurrent HCC after curative resection between January 2006 and December 2015 were considered eligible. Of them, 511 patients treated with TACE as the first-line anti-cancer treatment were selected. After excluding 83 patients according to our exclusion criteria, 428 patients were finally selected for the statistical analysis (**Fig 1**).

The baseline characteristics of 428 patients are summarized in **Table 1**. The median age of the study population was 59.2 (interquartile range [IQR], 52.2–66.6) years and male patients predominated (n = 362, 84.6%). Most patients had preserved liver function (Child-Pugh class A, n = 419, 97.9%), and the main etiology was hepatitis B virus infection (n = 348, 81.3%). The median AFP and des-gamma carboxyprothrombin (DCP) was 7.6 ng/mL and 31 mAU/mL, respectively. Around half of the study population had multiple tumors (n = 198, 46.3%), and portal vein invasion was identified in 26 (6.1%) patients. BCLC stage 0, A, B, and C HCC was observed in 109 (25.5%), 137 (32.0%), 68 (15.9%), and 114 (26.6%) patients, respectively. Multiple tumors and portal vein invasion were observed in 70 (16.4%), and 26 (6.1%) patients, and the median maximal tumor size was 3.5 (IQR, 2.5–5.2) cm at the time of resection. The median duration from resection to the first TACE was 17.9 (IQR 7.2–37.1) months.

**Table 1:** Baseline characteristics of the study population at the time of 1st TACE due to recurrent HCC after curative resection (n = 428).

Variables	Values
Demographic variables	
Age, years	59.2 (52.2–66.6)
Male gender	362 (84.6)
Diabetes mellitus	84 (19.6)
Ultrasonographic cirrhosis	178 (41.6)
Histological cirrhosis	263 (61.4)
Child-Pugh class, A/B	419 (97.9)/ 9 (2.1)
Etiology	
HBV/ HCV/ alcohol/ others	348 (81.3)/ 36 (8.4) / 13 (3.0) / 31 (7.2)
Laboratory variables	
Alanine aminotransferase, IU/L	26 (18–37)
Serum albumin, g/dL	4.0 (3.7–4.3)
Total bilirubin, mg/dL	0.8 (0.6–1.0)
Prothrombin time, INR	1.01 (0.97–1.06)
Platelet count, x10^3^/mm^3^	127 (96–165)
Alpha-fetoprotein, ng/mL	7.6 (3.0–74.5)
Des-gamma carboxyprothrombin, mAU/mL	31 (20–69)
Tumor variables	
Multiple tumors	198 (46.3)
Maximal tumor size, cm	1.6 (1.2–2.2)
Infiltrative pattern	8 (1.9)
Portal vein invasion	26 (6.1)
BCLC stage	
0/ A/ B/ C	109 (25.5) / 137 (32.0) / 68 (15.9) / 114 (26.6)
Tumor variables at the time of resection	
Multiple tumors	70 (16.4)
Maximal tumor size, cm	3.5 (2.5–5.2)
Portal vein invasion	32 (7.5)
Time from resection to the 1st TACE, months	17.8 (7.2–37.1)

Variables are expressed as n (%) or median (interquartile range).

TACE, transarterial chemoembolization; HCC, hepatocellular carcinoma; HBV, Hepatitis B virus; HCV, hepatitis C virus; INR, international normalized ratio; BCLC, Barcelona clinic liver cancer.

### Comparison between patients treated with TACE according to time of HCC recurrence

When our study population was divided into 2 groups with early (≤ 2 years, n = 255, 59.6%) and late (> 2 years, n = 173, 40.4%) recurrence [22], patients with early recurrence were significantly younger (median 57.8 vs. 62.0 years) and had a significantly higher incidence of diabetes (23.1% vs. 14.5%), a higher DCP level (median 33 vs. 27 mAU/mL), a higher incidence of multiple tumors (56.1% vs. 31.8%), a lower incidence of BCLC stage 0-A HCC (49.0% vs. 69.9%), and a higher maximal tumor size at the time of recurrence than those with late recurrence (all *P*<0.05) (**Table 2**).

**Table 2:** Comparison between patients treated with TACE due to early (≤ 2 years) and late (> 2 years) recurrence after curative resection.

Variables	Patients with early recurrence	Patients with late recurrence	*P* value
	(n = 255, 59.6%)	(n = 173, 40.4%)	
Demographic variables			
Age, years	57.8 (50.9–65.5)	62.0 (54.4–68.0)	<0.001
Male gender	212 (83.1)	150 (86.7)	0.316
Diabetes mellitus	59 (23.1)	25 (14.5)	0.026
Ultrasonographic cirrhosis	94 (36.9)	84 (48.6)	0.273
Histological cirrhosis	158 (62.0)	105 (60.7)	0.792
Child-Pugh class, A/B	249 (97.7)/ 6 (2.4)	170 (98.3)/ 3 (1.7)	0.745
Viral etiology	226 (88.7) / 29 (11.3)	158 (91.3) / 15 (8.7)	0.366
Laboratory variables			
Alanine aminotransferase, IU/L	26 (20–38)	23 (17–33)	0.199
Serum albumin, g/dL	3.9 (3.6–4.2)	4.1 (3.7–4.3)	0.059
Total bilirubin, mg/dL	0.7 (0.6–1.0)	0.8 (0.6–1.1)	0.074
Prothrombin time, INR	1.02 (0.97–1.07)	1.00 (0.96–1.06)	0.122
Platelet count, x10^3^/mm^3^	127 (96–163)	128 (96–166)	0.601
Alpha-fetoprotein, ng/mL	10.9 (3.6–104.4)	5.0 (2.7–27.6)	0.166
Des-gamma carboxyprothrombin, mAU/mL	33 (21–78)	27 (18–57)	0.043
Tumor variables			
Multiple tumors	143 (56.1)	55 (31.8)	<0.001
Maximal tumor size, cm	1.5 (1.1–2.1)	1.6 (1.3–2.3)	0.205
Infiltrative pattern	5 (2.0)	3 (1.7)	0.846
Portal vein invasion	13 (5.1)	13 (7.5)	0.304
BCLC stage, 0-A/ B-C	125 (49.0) / 130 (51.0)	121 (69.9) / 52 (30.1)	<0.001
Tumor variables at the time of resection			
Multiple tumors	47 (18.4)	23 (13.3)	0.160
Maximal tumor size, cm	4.0 (2.7–5.8)	3.1 (2.2–4.5)	0.001
Portal vein invasion	21 (8.2)	11 (6.4)	0.471

Variables are expressed as n (%) or median (interquartile range).

TACE, transarterial chemoembolization; INR, international normalized ratio; BCLC, Barcelona clinic liver cancer.

### Progression to BCLC stage C during repetitive TACEs

**Fig 2:**
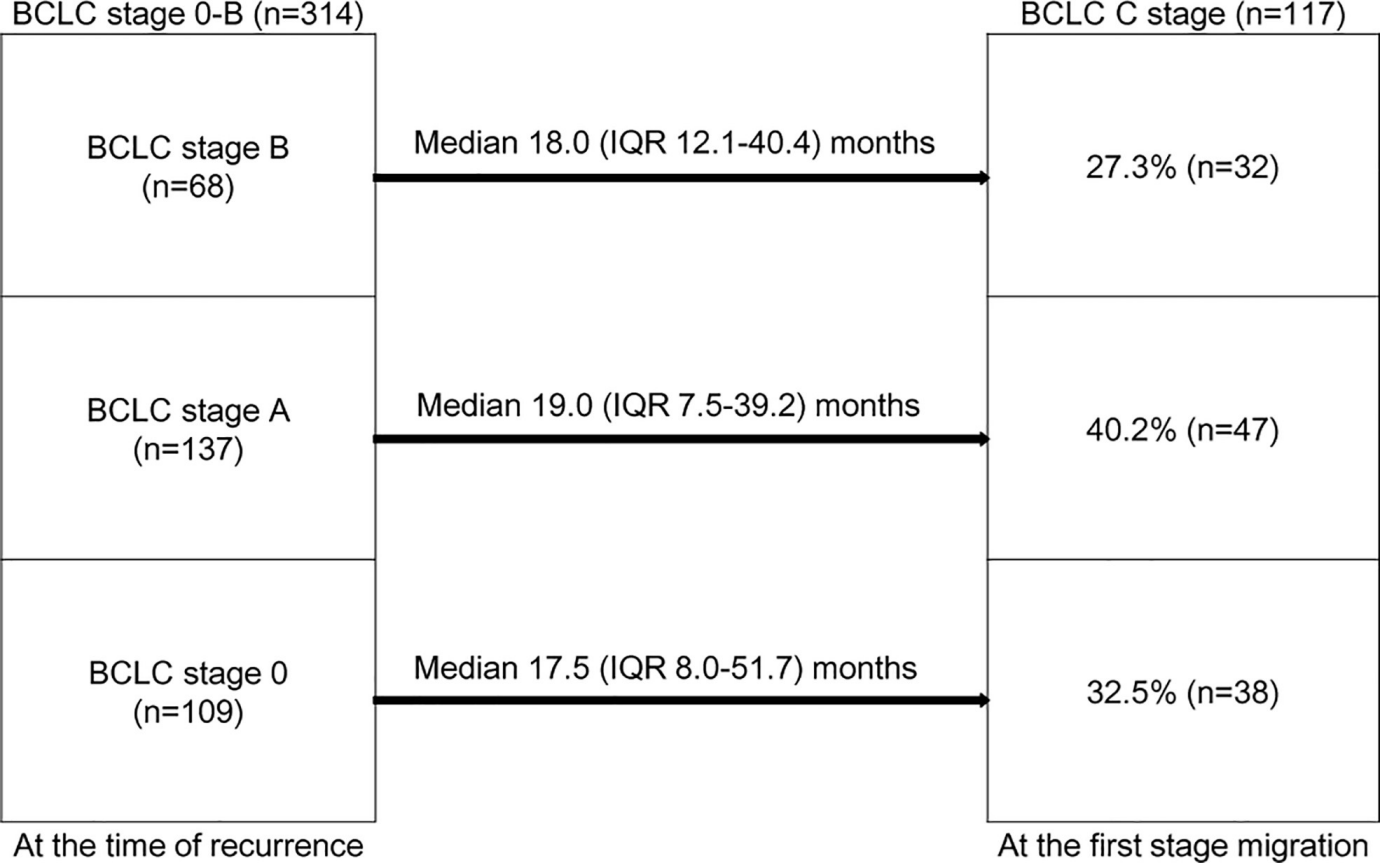
Distribution of progression among patients with Barcelona Clinic Liver Cancer (BCLC) stage C among patients with BCLC stage 0-B. Among patients with BCLC stage 0 at the time of recurrence, 38 patients with BCLC stage 0, 47 patients with BCLC stage A, and 32 patients with BCLC stage B experienced progression to BCLC stage C.

A total of 314 patients had BCLC stage 0-B HCC at the time of recurrence (109 with BCLC stage 0, 137 with BCLC stage A, and 68 with BCLC stage B). Among patients with BCLC stage 0 at the time of recurrence, 38 (32.5%) experienced progression to BCLC stage C after a median follow-up of 17.5 (IQR 8.0–51.7) months. In addition, 47 (40.2%) patients with BCLC stage A and 32 (27.3%) patients with BCLC stage B at the time of recurrence progressed to BCLC stage C after a median follow-up of 19.0 (IQR 7.5–39.2) and 18.0 (IQR 12.1–40.4) months, respectively (**Fig 2**).

### Predictors of progression to BCLC stage C (BCLC stage 0-B only)

On univariate analysis, DCP level, and ≥2 TACEs within 6 months were significantly associated with progression to BCLC stage C in patients with BCLC stage 0-B only (all *P*<0.05) (**Table 3**). On multivariate analyses, DCP level and ≥2 TACEs within 6 months were independently associated with a higher risk of progression to BCLC stage C (HR = 1.000, 95% CI 1.000–1.001, *P*<0.001; HR = 1.898, 95% CI 1.265–2.846, *P* = 0.002, respectively) (**Table 3**).

**Table 3:** Independent predictors of stage progression to BCLC stage C among patients with recurrent HCC after curative resection (BCLC 0-B only).

Variables	Univariate	Multivariate analysis		
	*P* value	HR	95% CI	*P* value
At the time of recurrence				
Age	0.073			
Male gender	0.532			
Diabetes mellitus	0.677			
Alanine aminotransferase, IU/mL	0.063			
Serum albumin, g/dL	0.059			
Total bilirubin, mg/dL	0.996			
Prothrombin time, INR	0.875			
Platelet count, x10^3^/mm^3^	0.765			
Alpha-fetoprotein > 100, ng/mL	0.376			
Des-gamma carboxyprothrombin, mAU/mL	<0.001	1.000	1.000–1.001	<0.001
Multiple tumors	0.170			
Maximal tumor size >5cm	0.745			
More than 2 TACEs within 6 months	0.003	1.898	1.265–2.846	0.002
More than 3 TACEs within 6 months	0.051			
At the time of resection				
Multiple tumors	0.407			
Maximal tumor size >5cm	0.177			

BCLC, Barcelona clinic liver cancer; HCC, hepatocellular carcinoma; HR, hazard ratio; CI, confidence interval; INR, international normalized ratio; TACE, trans-arterial chemoembolization.

More than 2 TACEs within 6 months showed a borderline statistical significance in the subgroup with early recurrence (≤ 2 years, n = 176, 56.1%) (*P* = 0.053), whereas it was not significant in the subgroup with late recurrence (>2 years, n = 138, 43.9%) (*P*>0.05) (**Table 4**).

**Table 4:** Independent predictors of stage-progression in patients with early and late HCC recurrence after curative resection (BCLC 0-B only).

Variables	≤ 2-years (n = 176, 56.1%)				> 2-years (n = 138, 43.9%)			
	Univariate	Multivariate analysis			Univariate	Multivariate analysis		
	*P* value	HR	95% CI	*P* value	*P* value	HR	95% CI	*P* value
At the time of recurrence								
Age	0.991				0.409			
Male gender	0.609				0.388			
Diabetes mellitus	0.995				0.790			
Alanine aminotransferase, IU/mL	0.274				0.390			
Serum albumin, g/dL	0.115				0.764			
Total bilirubin, mg/dL	0.752				0.350			
Prothrombin time, INR	0.257				0.441			
Platelet count, x10^3^/mm^3^	0.807				0.920			
Alpha-fetoprotein > 100, ng/mL	0.578				0.134			
Des-gamma carboxyprothrombin, mAU/mL	0.001	1.000	1.000–1.001	0.001	0.049	1.002	1.000–1.005	0.089
Multiple tumors	0.369				0.986			
Maximal tumor size >5cm	0.586				0.019	7.748	0.950–63.201	0.056
More than 2 TACEs within 6 months	0.053				0.317			
More than 3 TACEs within 6 months	0.369				0.751			
At the time of resection								
Multiple tumors	0.485				0.847			
Maximal tumor size >5cm	0.233				0.961			

HCC, hepatocellular carcinoma; BCLC, Barcelona clinic liver cancer; HR, hazard ratio; CI, confidence interval; INR, international normalized ratio; TACE, transarterial chemoembolization.

### Predictors of mortality

In the entire cohort, on univariate analyses, lower serum albumin level, higher prothrombin time, AFP level >100 ng/mL, multiple tumors, and ≥2 or ≥3 TACEs within 6 months were significantly associated with a higher risk of mortality (all *P*<0.05) (**Table 5**). On multivariate analysis using ≥2 TACEs, ≥2 TACEs within 6 months independently predicted a higher risk of mortality (HR = 1.863, 95% CI 1.372–2.530, *P*<0.001), together with lower serum albumin level, AFP level >100 ng/mL, and multiple tumors (all *P*<0.05) (**Table 5**). In addition, on multivariate analysis of ≥3 TACEs, ≥3 TACEs within 6 months independently predicted a higher risk of mortality (HR = 1.620, 95% CI 1.029–2.551, *P* = 0.037), together with lower serum albumin level, AFP level >100 ng/mL, and multiple tumors (all *P*<0.05) (**Table 5**).

**Table 5:** Independent predictors of mortality among patients with recurrent HCC after curative resection.

Variables	Univariate	Multivariate analysis*			Multivariate analysis**		
	*P* value	HR	95% CI	*P* value	HR	95% CI	*P* value
At the time of recurrence							
Age	0.247						
Male gender	0.961						
Diabetes mellitus	0.545						
Alanine aminotransferase, IU/mL	0.446						
Serum albumin, g/dL	<0.001	0.547	0.391–0.767	<0.001	0.538	0.384–0.755	<0.001
Total bilirubin, mg/dL	0.900						
Prothrombin time, INR	<0.001	3.713	0.916–15.054	0.066	3.700	0.906–15.108	0.068
Platelet count, x10^3^/mm^3^	0.205						
Alpha-fetoprotein > 100, ng/mL	0.015	1.495	1.071–2.087	0.018	1.527	1.092–2.136	0.013
Des-gamma carboxyprothrombin, mAU/mL	0.287						
Multiple tumors	<0.001	1.569	1.174–2.097	0.002	1.597	1.189–2.145	0.002
Maximal tumor size >5cm	0.104						
More than 2 TACEs within 6 months	<0.001	1.863	1.372–2.530	<0.001			
More than 3 TACEs within 6 months	0.001				1.620	1.029–2.551	0.037
At the time of resection							
Multiple tumors	0.448						
Maximal tumor size >5cm	0.644						

HCC, hepatocellular carcinoma; HR, hazard ratio; CI, confidence interval; INR, international normalized ratio; TACE, transarterial chemoembolization.

Multivariate analysis* and ** includes ≥2 and ≥3 TACEs within 6 months as a variable for multivariate analysis, respectively.

In the subgroup with early recurrence (≤ 2 years), ≥2 TACEs within 6 months independently predicted a higher risk of mortality (HR = 1.629, 95% CI 1.134–2.340, *P* = 0.008), together with lower serum albumin level and multiple tumors (all *P*<0.05), whereas ≥3 TACEs within 6 months was not significant in univariate analysis (*P* = 0.075) (**S1 Table**). In the subgroup with late recurrence (>2 years), ≥2 and ≥3 TACEs within 6 months were independently associated with increased risk of mortality (HR = 2.386, 95% CI 1.268–4.488, *P* = 0.007 and HR = 9.297, 95% CI 2.087–41.405, *P* = 0.003, respectively), together with lower serum albumin level, higher prothrombin time, and higher DCP level (all *P*<0.05) (**S1 Table**).

### Progression-free survival rate to BCLC stage C (BCLC 0-B only) and overall survival rate

**Fig 3:**
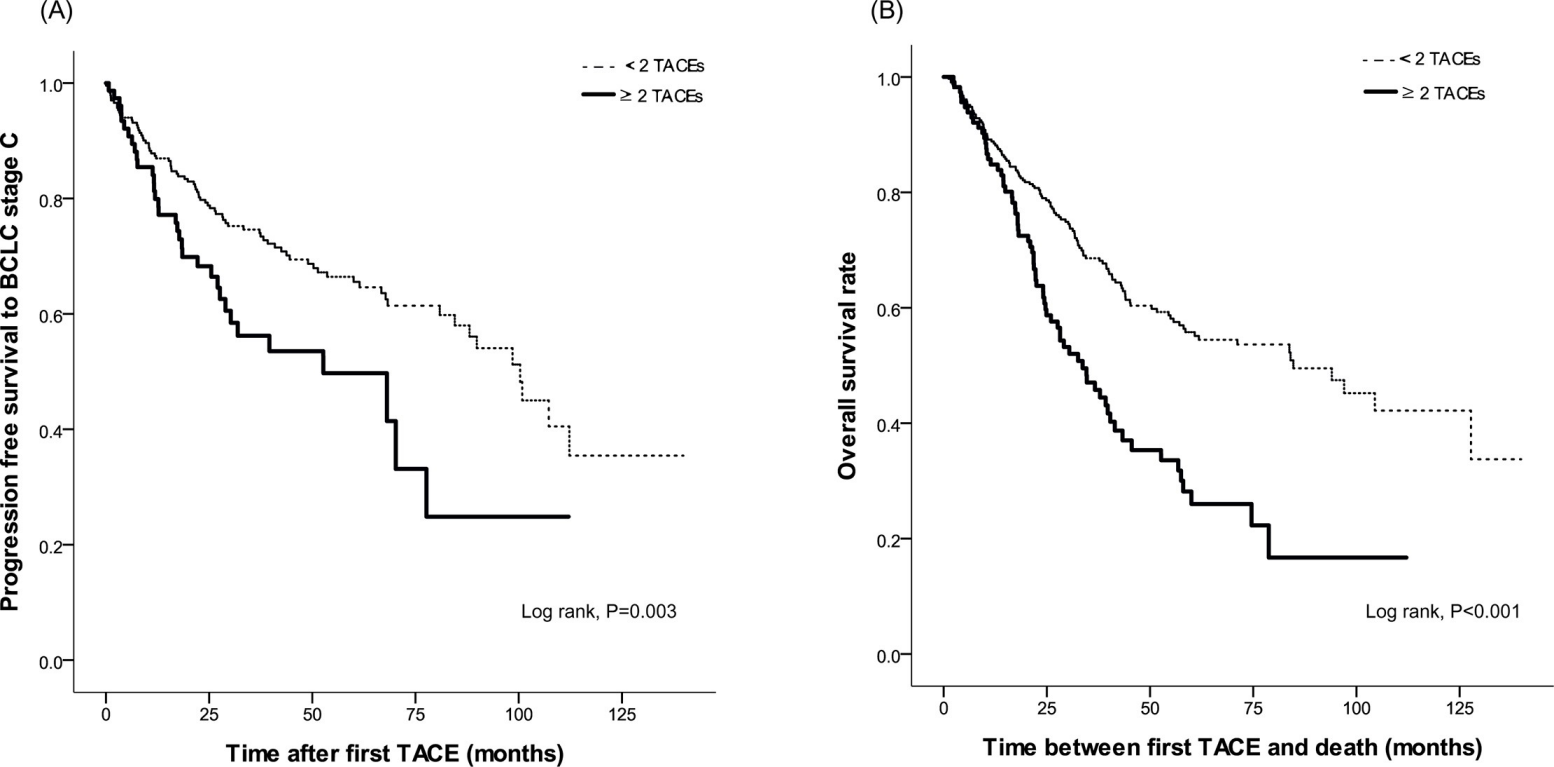
**Cumulative incidence rates of progression to Barcelona Clinic Liver Cancer (BCLC) stage C among patients with BCLC stage 0-B (A) and mortality in the entire population (B) with ≥2 and <2 TACEs within 6 months** (all *P*<0.05, log-rank test).

The progression-free survival to BCLC stage C were significantly associated with between <2 and ≥2 TACEs within 6 months in patients with only BCLC stage 0-B (*P* = 0.003, log-rank test), and the overall survival rates were significantly associated with between <2 and ≥2 TACEs within 6 months in the entire cohort (*P*<0.001, log-rank test) (**Fig 3**). The progression-free survival rates at 5-years were 65.6% for <2 TACEs and 49.7% for ≥2 TACEs within 6 months in patients with only BCLC stage 0-B, and the overall survival rates at 5-years, were 55.8% for <2 TACEs and 26.0% for ≥2 TACEs within 6 months in the entire cohort (**Fig 3**).

## Discussion

In this study, we found that ≥2 TACEs within 6 months because of recurrent HCC after curative resection independently predicted progression to BCLC stage C in the subgroup of patients with BCLC stage A or B at the time of recurrence (HR = 1.898). In addition, ≥2 and ≥3 TACEs within 6 months were independently associated with increased risk of mortality (HR = 1.863 and 1.620, respectively), and ≥2 TACEs independently predicted mortality regardless of the recurrence time point (HR = 1.629 in the subgroup with early recurrence and HR = 2.386 in the subgroup with late recurrence). The presence of ≥3 TACEs was also independently predictive of mortality in the subgroup with late recurrence (HR = 9.297).

It is well known that TACE provides survival benefit for patients with intermediate-stage HCC, defined as large, unresectable or multinodular HCC [10, 23]. However, because the primary aim of TACE is palliative management, it should be carefully performed to prolong survival in the presence of TACE-associated morbidity and mortality. Indeed, TACE may worsen the underlying liver dysfunction, especially when repeated TACEs are required. Moreover TACE has an up to 10% risk rate of potential procedure-related mortality [14]. Before the era of molecular targeted treatments, TACE has been frequently repeatedly performed due to the absence of alternative treatment modalities, even when poor patient response was anticipated [14]. However, because several molecular targeted agents, including sorafenib, are now available based on their proven treatment efficacy from several randomized trials [24], the concept of TACE refractoriness was proposed to prevent the deterioration of liver function or stage progression during repeated TACE sessions [15, 24–26].

The Japan Society of Hepatology (JSH) and the Liver Cancer Study Group of Japan in 2010 introduced the concept of TACE refractoriness (≥ 2 ineffective responses of the treated tumor [viable lesions >50%] or ≥ 2 progressive increases in total tumor counts, continuous elevation of the levels of tumor markers, or appearance of vascular invasion extrahepatic spread) [27, 28]. Because the change in arterial enhancement of HCC should be repeatedly assessed after each TACE session to define TACE refractoriness [29], the JSH criteria for TACE refractoriness might be inconvenient for hepatologists or oncologists. In contrast, Kim et al. [14] defined TACE refractoriness according to the number of TACE sessions within a limited period (≥ 3 TACEs within 6 months), which was significantly associated with an increased risk of stage progression and accordingly was incorporated into the Korean Association for the Study of the Liver guideline [16]. This criterion for TACE refractoriness based on the number of TACE sessions with in a restricted period might be easy to use in clinical practice.

Although several factors were significantly different between patients with early recurrence and those with late recurrence in our study, ≥ 2 TACE within 6 months for recurrent HCC after curative resection independently predicted progression to BCLC stage C among patients with BCLC stager 0-B at the time of the first TACE. In addition, the progression free survival to BCLC stage C was significantly different between the subgroups with <2 TACEs and ≥2 TACEs within 6 months. However, probably due to the relatively small sample size of the subgroups with early and late recurrence, the independent influence of ≥ 2 TACEs within 6 months was not reproduced in the subgroup analysis. In contrast to our study, Kim et al. reported ≥ 3 TACEs within 6 months as an independent predictor of stage progression [14]. Although the reason for this discrepancy is not clear, it can be explained in part by the different characteristics between treatment-naïve patients who started anti-cancer treatment with TACE and those treated with TACE due to recurrent HCC after curative resection.

In patients who underwent curative resection, HCC recurrence might be detected in the early stage probably because of the intensive surveillance involving frequent dynamic imaging studies. Indeed, in our study population, the median maximal tumor size was less than 2cm and around three-fourths of the patients had BCLC stage 0-B HCC at the time of recurrence. In addition, patients who underwent surgical resection might have well-preserved liver function preoperatively. However, a pre-existing history of HCC treatment itself (resection) and the potential vulnerability to liver deterioration due to repeated TACEs, because of reduced liver volume after resection might be associated with the fewer requited TACE sessions to define TACE refractoriness in patients with recurrent HCC after resection. Similarly, the association between ≥ 2 TACEs within 6 months and the increased risk of mortality can be explained.

Our study has several strengths. First, this study has a relatively large sample size, with a long-term follow-up period (median 32.1 months, up to 59.3 months). These factors might have increased the statistical power of our study and enabled us to identify poor prognostic factors for long-term prognosis in our cohort. Second, to the best of our knowledge, this study is the first to identify the prognostic factors of stage progression and mortality and to establish “TACE refractoriness” optimized for patients who underwent TACE because of recurrent HCC after resection. Third, although discrepant characteristics or pathophysiologies might exist, we also confirmed that the term “TACE refractoriness” might be adaptable, regardless of the time point of HCC recurrence.

Several issues remain unresolved in our study. First, our study was a single center, retrospective study. Although our study has a large sample size and long-term follow-up period, with strong statistical power, our results need to be further validated in a large-scale prospective study. Second, in case of TACE refractoriness, the proportion of patients who received sorafenib was extremely low; thus, further prospective, randomized control studies investigating whether switching to sorafenib, based on the concept of TACE refractoriness proposed in our study, can provide a survival benefit are strongly required. Third, our study mainly focused on the number of TACE sessions without considering the reasons why repeated TACEs were required, such as incomplete previous TACE or the development of new lesions. As the presence of ≥ 2 ineffective responses seen in > 50% according to the modified RECIST criteria is defined as TACE refractoriness in the JSH guideline [28], further studies are needed to validate whether the prognostic value of our simple definition of TACE refractoriness would be comparable to that of the JSH.

In conclusion, the progression-free survival rates and overall survival rates in patients treated with repeated TACEs due to recurrent HCC after resection were poor in patients who underwent ≥ 2 TACEs. Thus, ≥ 2 TACEs within 6 months could also be defined as TACE refractoriness in our cohort of patients with recurrent HCC after curative resection who were treated with TACE. However, whether maintaining repeated TACEs or switching to the next-step treatment, such as molecular targeted agents, would provide a better survival benefit should further be investigated.

## Supporting information

S1 Dataset Spreadsheet with the minimal data set underlying the results. (XLSX)

S1 Table Independent predictors of mortality among patients with early and late HCC recurrence after curative resection. (DOCX)

## Abbreviations

HCC: hepatocellular carcinoma
TACE: trans-arterial chemoembolization
CT: computed tomography
MRI: magnetic resonance imaging
BCLC: Barcelona Clinic Liver Cancer
AFP: alpha-fetoprotein
RECIST: Response Evaluation Criteria in Solid Tumors
HR: hazard ratio
CI: confidence interval
IQR: interquartile range
DCP: Des-gamma carboxyprothrombin
JSH: Japan Society of Hepatology
