# Supplementary material for: Refractoriness to transarterial chemoembolization in patients with recurrent hepatocellular carcinoma after curative resection
Source: PLoS One. 2019 Apr 4;14(4):e0214613. doi: 10.1371/journal.pone.0214613 (PMC6449032; doi:10.1371/journal.pone.0214613)
Supplement: S1 Table — (DOCX) [file pone.0214613.s002.docx]

| **Supplementary Table 1. Independent predictors of mortality among patients with early and late HCC recurrence after curative resection** | | | | | | | | | | | | | | | |
| --- | --- | --- | --- | --- | --- | --- | --- | --- | --- | --- | --- | --- | --- | --- | --- |
| Variables | ≤ 2-years (n=255, 59.6%) | | | | |  | > 2-years (n=173, 40.4%) | | | | | | | | |
|  | Univariate |  | Multivariate analysis* | | |  | Univariate |  | Multivariate analysis* | | |  | Multivariate analysis** | | |
|  | *P* value |  | HR | 95% CI | *P* value |  | *P* value |  | HR | 95% CI | *P* value |  | HR | 95% CI | *P* value |
| At the time of recurrence |  |  |  |  |  |  |  |  |  |  |  |  |  |  |  |
| Age | 0.551 |  |  |  |  |  | 0.887 |  |  |  |  |  |  |  |  |
| Male gender | 0.716 |  |  |  |  |  | 0.908 |  |  |  |  |  |  |  |  |
| Diabetes mellitus | 0.185 |  |  |  |  |  | 0.809 |  |  |  |  |  |  |  |  |
| Alanine aminotransferase, IU/mL | 0.527 |  |  |  |  |  | 0.071 |  |  |  |  |  |  |  |  |
| Serum albumin, g/dL | <0.001 |  | 0.554 | 0.381 - 0.806 | 0.002 |  | 0.002 |  | 0.495 | 0.265 - 0.922 | 0.027 |  | 0.449 | 0.236 - 0.854 | 0.015 |
| Total bilirubin, mg/dL | 0.443 |  |  |  |  |  | 0.058 |  |  |  |  |  |  |  |  |
| Prothrombin time, INR | 0.107 |  |  |  |  |  | <0.001 |  | 84.338 | 3.946 - 1802.480 | 0.005 |  | 44.771 | 2.017 - 991.077 | 0.016 |
| Platelet count, x10^3^/mm^3^ | 0.371 |  |  |  |  |  | 0.458 |  |  |  |  |  |  |  |  |
| Alpha-fetoprotein > 100, ng/mL | 0.091 |  |  |  |  |  | 0.687 |  |  |  |  |  |  |  |  |
| Des-gamma carboxyprothrombin, mAU/mL | 0.660 |  |  |  |  |  | <0.001 |  | 1.003 | 1.002 - 1.004 | <0.001 |  | 1.003 | 1.002 - 1.004 | <0.001 |
| Multiple tumors | 0.002 |  | 1.463 | 1.020 - 2.100 | 0.039 |  | 0.620 |  |  |  |  |  |  |  |  |
| Maximal tumor size >5cm | 0.865 |  |  |  |  |  | 0.003 |  | 2.835 | 0.692 - 11.621 | 0.148 |  | 3.215 | 0.792 - 13.046 | 0.102 |
| More than 2 TACEs within 6 months | 0.001 |  | 1.629 | 1.134 - 2.340 | 0.008 |  | 0.024 |  | 2.386 | 1.268 - 4.488 | 0.007 |  | - | - | - |
| More than 3 TACEs within 6 months | 0.075 |  | - | - | - |  | 0.022 |  | - | - | - |  | 9.297 | 2.087 - 41.405 | 0.003 |
| At the time of resection |  |  |  |  |  |  |  |  |  |  |  |  |  |  |  |
| Multiple tumors | 0.923 |  |  |  |  |  | 0.448 |  |  |  |  |  |  |  |  |
| Maximal tumor size >5cm | 0.237 |  |  |  |  |  | 0.644 |  |  |  |  |  |  |  |  |
| HR, hazard ratio; CI, confidence interval; INR, international normalized ratio; TACE, transarterial chemoembolization. | | | | | | | | | | | | | | | |
| Multivariate analysis* and ** includes ≥2 and ≥3 TACEs within 6 months as a variable for multivariate analysis, respectively. | | | | | | | | | | | | | | | |

**Supporting information**

**S1 dataset.** Spreadsheet with the minimal data set underlying the results
